# Chromosomal Macrodomains and Associated Proteins: Implications for DNA Organization and Replication in Gram Negative Bacteria

**DOI:** 10.1371/journal.pgen.1002123

**Published:** 2011-06-16

**Authors:** Remus T. Dame, Olga J. Kalmykowa, David C. Grainger

**Affiliations:** 1 Leiden Institute of Chemistry, Gorlaeus Laboratories, Laboratory of Molecular Genetics and Cell Observatory, Leiden University, Leiden, The Netherlands; 2 School of Life Sciences, University of Warwick, Coventry, United Kingdom; Agency for Science, Technology, and Research,

## Abstract

The *Escherichia coli* chromosome is organized into four macrodomains, the function and organisation of which are poorly understood. In this review we focus on the MatP, SeqA, and SlmA proteins that have recently been identified as the first examples of factors with macrodomain-specific DNA-binding properties. In particular, we review the evidence that these factors contribute towards the control of chromosome replication and segregation by specifically targeting subregions of the genome and contributing towards their unique properties. Genome sequence analysis of multiple related bacteria, including pathogenic species, reveals that macrodomain-specific distribution of SeqA, SlmA, and MatP is conserved, suggesting common principles of chromosome organisation in these organisms. This discovery of proteins with macrodomain-specific binding properties hints that there are other proteins with similar specificity yet to be unveiled. We discuss the roles of the proteins identified to date as well as strategies that may be employed to discover new factors.

## Introduction

**Figure 1:**
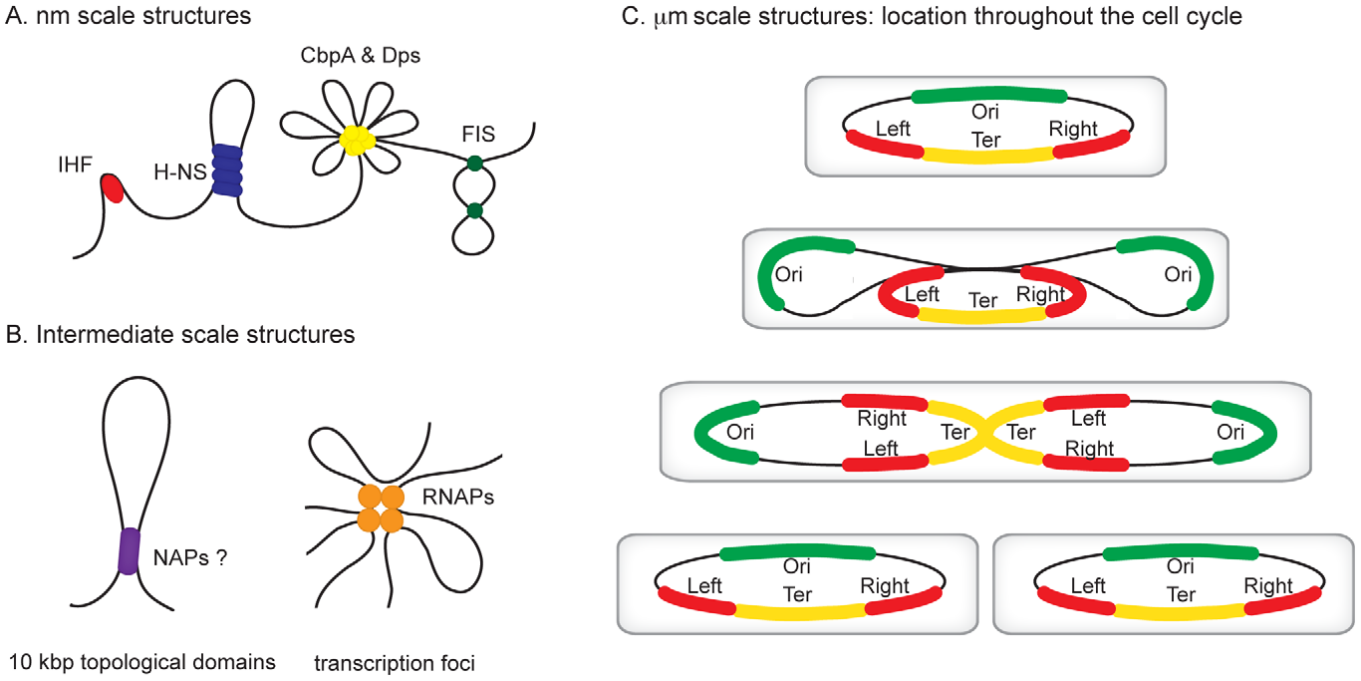
Hierarchical levels of organization in bacterial chromosomes. Different levels of organization exist within bacterial chromosomes. (A) At the nm scale nucleoid proteins such as HU, H-NS, CbpA, Dps, and Fis organize the genome by driving events such as DNA bending, bridging, and aggregation. (B) Structures such as seen in (A) likely exist within, and may contribute towards the formation of looped topological domains (on average each ∼10 kbp in size) and transcription foci, where multiple transcribing RNA polymerase molecules are clustered potentially also yielding loops along the genome. (C) All of the above could add to the complexity of the organization within individual macrodomains. The individual macrodomains have a defined localization within the cell throughout the cell cycle. In newborn cells *ori* and *ter* are located at mid-cell positions. These sites are located centrally within the Ori and Ter macrodomains. The Left and Right macrodomains occupy positions close to the cell poles. Upon replication, the Ori domains move towards the cell poles. Right before cell division the replicated Ter domains segregate. The chromosome in the daughter cells has again the same Left-Right orientation. MatP preferentially occupies sites in the Ter domain, whereas SlmA and SeqA are absent from this domain.

All organisms are faced with the challenge of organising their genetic content within the confines of the cell or its compartments. In eukaryotes, DNA is packed inside the nucleus and histone proteins are known to wrap DNA into nucleosomes. Nucleosomal arrays are folded into chromatin fibers, which are themselves folded into higher order structures. Whilst our understanding of this process at the nucleosomal level is well developed, higher levels of organization are poorly understood [1], [2]. Similarly, mechanisms of chromosome organisation in bacteria are poorly defined. The folded bacterial genome, or nucleoid, is known to be organized by “nucleoid-associated” DNA-binding proteins (NAPs), DNA supercoiling, and transcription [3]. Nucleoid-associated proteins are abundant, often bind DNA with a low degree of sequence specificity, and impose constraints on DNA topology that are best understood at the nm scale (Figure 1A). For example histone-like nucleoid structuring protein (H-NS) can stimulate DNA-bridging events, the integration host factor (IHF) can introduce hair-pin bends into the double helix and curved DNA binding protein A (CbpA) forms aggregates with DNA [4]–[6]. It is likely that some of these nucleoid-associated proteins contribute to the formation of structures at larger scales, such as topologically isolated supercoiled domains and transcription foci (Figure 1B), but fine molecular details remain to be elucidated [7], [8]. In this review, we focus on recent observations concerning organisation of bacterial chromosomes into even larger organisational units at the µm scale: macrodomains (Figure 1C) [9]–[12]. In particular we focus on the implications of recent findings regarding three proteins—SeqA, SlmA, and macrodomain Ter protein (MatP)—with macrodomain-specific DNA-binding properties.

### Identification of the Chromosomal Macrodomains

**Figure 2:**
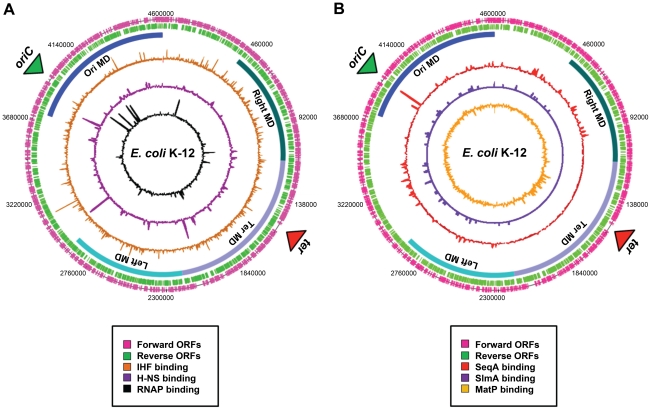
Distribution of nucleoid-associated proteins across the *E. coli* chromosome. (A) A genome atlas where ChIP-chip datasets [41] for IHF (orange), H-NS (purple), and RNA polymerase (black) are plotted against the features of the *E. coli* chromosome. (B) A genome atlas where ChIP-chip or ChIP-Seq datasets for SeqA (red) [17], SlmA [purple] (19) and MatP [orange] (20) are plotted against the features of the *E. coli* chromosome. The locations of ORFs are shown as pink and green lines. The positions of the four macrodomains (MDs) are shown as blue bars and are labelled.

Evidence for the existence of chromosomal “macrodomains” in *E. coli* has been established during the last 5 years by Boccard and coworkers [9], [13]–[15], building on the ideas of Niki et al. [10]. The existence and positioning of the four macrodomains was first determined in assays aimed at resolving spatial proximity of genomic regions by measuring the frequency of recombination between phage λ *att* sites scattered throughout the *E. coli* chromosome [13]. This analysis revealed a clear bias in the positioning of pairs of *att* sites that supported efficient recombination and thus were spatially close. On the basis of these observations, it was concluded that the *E. coli* chromosome is organized into four discrete structured subdomains and that *att* sites in each domain interact primarily with the *att* sites in the same domain. Each of these domains (Ori, Right, Left, and Ter) contains approximately 1 Mbp of DNA. The localization of the macrodomains is subject to changes during the cell cycle, but is fairly well defined (Figure 1C). The degree of linear DNA compaction as measured in vivo using genomic markers varies among domains. The 800-kb domain around Ter is on average five times less compact than the rest of the genome and extends between two opposing ends of the nucleoid [16]. The highly abundant nucleoid-associated proteins are obvious candidates for bestowing unique properties on the individual macrodomains. However, available evidence suggests that this is unlikely; well-characterised nucleoid-associated proteins such as H-NS and IHF are found to bind with all of the macrodomains in chromatin immunoprecipitation (ChIP) experiments (Figure 2A). Indeed, amongst the known drivers of chromosome structure, only RNA polymerase displays any domain-specific binding behaviour; its primary targets, the seven rRNA operons, are all in the *oriC* half of the chromosome (Figure 2A).

### Proteins with Macrodomain-Specific DNA-Binding Properties

High-throughput analysis of DNA-binding events across bacterial genomes using ChIP has revealed that some major regulators of the cell cycle have macrodomain-specific DNA-binding profiles [17]–[21]. MatP binds exclusively to the Ter macrodomain [20], whilst both SeqA and SlmA are excluded from this region of the chromosome [17]–[19], [21]. The fact that SeqA, SlmA, and MatP bind to nondegenerate DNA target sites with a high degree of specificity, sets them apart from the classical nucleoid-associated proteins [16], [19]–[21]. However, since the term “nucleoid-associated protein” is clearly ambiguous (discussed in [3]), we argue that it can be applied to any protein that plays a role in organising the chromosome. Thus, below we discuss the known properties of SeqA, SlmA, and MatP in light of their recently discovered macrodomain-specific chromosome-binding properties.

### SeqA

The SeqA protein was originally discovered as the factor responsible for sequestration of chromosome replication origins in bacteria [22]. It has subsequently been shown that SeqA plays a key role in preventing the over-initiation of chromosome replication [23] and delays the separation of new chromosomes [24]. SeqA recognises pairs of hemi-methylated GATC motifs that are found in newly replicated DNA. Whilst these motifs are most densely concentrated near *oriC*, many other potential SeqA targets are distributed across the chromosome. It has long been assumed that SeqA might bind hundreds of sites distal to *oriC*, and two ChIP studies recently confirmed these suspicions [17], [18]. Surprisingly, these studies also demonstrated that SeqA is excluded from the Ter macrodomain except under artificial conditions where chromosome replication is blocked (Figure 2B) [17]. This exclusion is most likely due to a lack of high affinity SeqA binding sites in the Ter macrodomain [17]. SeqA is known to associate with the cell membrane and, given the skewed binding of SeqA across the genome, SeqA may play a role to properly orientate the chromosome during cell division. Due to changes in the methylation state of the DNA as the chromosome is replicated, the SeqA distribution across the genome is dynamic. These changes may influence the structure and/or cellular position of the Ori, Right, and Left macrodomains as the chromosome is copied. It is unknown if the process of DNA replication affects SlmA or MatP binding but, as outlined below, all three proteins are known to play key roles in controlling chromosome replication and separation.

### SlmA

**Figure 3:**
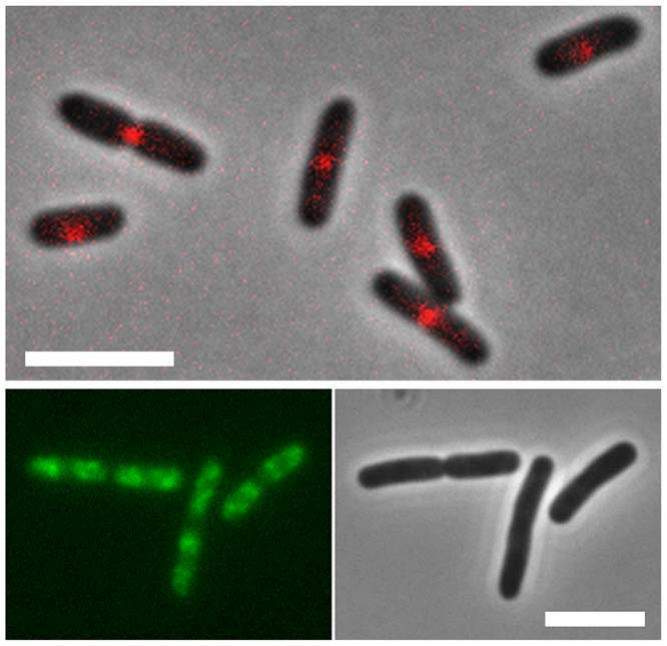
Localization of MatP and SlmA on the *E. coli* chromosome. *E. coli* cells expressing fluorescent derivatives of matP (matP-Cherry) (top panel) and SlmA (GFP-SlmA) (bottom panel). An overlay of phase contrast and fluorescence images is shown for matP, whereas separate fluorescence and DIC images are shown for SlmA. Scale bar, 4 µm. MatP predominantly localizes to the Ter macrodomain, whereas SlmA is absent from this domain.

The SlmA protein was identified in genetic screens as a “nucleoid occlusion” factor, i.e., as a protein involved in coordinating positioning and proper assembly of the so-called Z-ring at mid-cell prior to cell division [25]. The assembly of the Z-ring relies on the multimerization of the tubulin-like FtsZ protein, to which subsequently other septal ring components are recruited. The molecular basis underlying the action of SlmA was recently investigated in two parallel studies [19], [21]. These studies showed that SlmA can bind DNA and simultaneously interact with FtsZ, interfering with Z-ring assembly [19], [21]. Genome-wide ChIP showed that SlmA binds to a 12-bp palindromic consensus sequence (GTGAGTACTCAC), which is found 50 times along the *E. coli* K-12 genome. Strikingly, none of these sites are found in the Ter macrodomain and they are underrepresented in the Left and Right macrodomains (Figure 2B). Sequence analysis reveals that putative SlmA binding sites are also excluded from the Ter macrodomain of pathogenic *E. coli* strains, *Salmonella Typhimurium*, and *Klebsiella pneumoniae* [19]. The unique presence of SlmA binding sites in non-Ter domains suggests a model in which SlmA bound in these genomic regions prevents undesired Z-ring formation, whilst permitting Z-ring formation at Ter-sites that prior to cell division are located at mid-cell (Figure 3) [26]. One might speculate that the FtsZ-SlmA structures that are nonproductive for Z-ring formation act in contributing to a structural framework to which the nucleoid is tethered. SlmA works together with the MinCDE system in ensuring that the cytokinetic ring is properly positioned. MinCDE prevents cells from dividing near the poles and promotes the positioning of the cytokinetic ring near midcell, while SlmA prevents the premature assembly of the cytokinetic ring over unsegregated chromosomes [21], [27]. Although this review is focused on the *E. coli* system, it is pertinent to note that proteins similar in function to SlmA have been identified in other bacteria. Thus, the nucleoid occlusion protein Noc of *Bacillus subtilis* also acts as a spatial regulator of cell division by binding to sites outside the terC region of the chromosome [28]. The MipZ protein appears to play a similar role in *Caulobacter*. Owing to its interaction with ParB, which binds specifically to the origin region, upon origin segregation MipZ localizes to the poles where it destabilizes the polar FtsZ complex and directs FtsZ polymerization towards midcell [29].

### MatP

MatP is a small DNA-binding protein that—unlike SeqA and SlmA—is associated exclusively with the Ter domain of the *E. coli* genome (Figure 3) [20]. It binds specifically to a signature motif of 13 bps (GTGACA/GNT/CGTCAC) repeated 23 times within the Ter region. It is intriguing to note that the flanking four bps of the binding site of MatP and that of SlmA are identical. The MatP binding motif (*matS),* was discovered in silico by searching for scattered domain-specific targets of nucleoid-associated proteins. The factor specifically binding to this site (MatP) was identified in DNA-binding assays using crude *E. coli* extracts [20] as the product of the *ycbG* gene. The high affinity binding of MatP within the Ter domain was visualized in vivo using fluorescent microscopy. These experiments showed that MatP prevents premature chromosome segregation early during the cell cycle by keeping the Ter regions of two chromosomes together. In MatP knock-out cells this prolonged colocalization of the Ter domains is not observed. Fast growing cells deficient in MatP display a filament-like or anucleate phenotype. A delay in segregation of the daughter chromosomes due to the binding of MatP to the Ter region thus appears essential in coordinating chromosome segregation and cell division. Also, without MatP, the Ter domain displays higher mobility and a lower degree of compaction. Surprisingly the effects of MatP-DNA binding stretch over long distances. The deletion of a *matS* site increases the mobility of regions even several tens of kb away. While the role of this protein in the cell cycle and the organization of the Ter domain is apparent, the mechanism of MatP action is still unknown. Two models have been proposed for how MatP organizes the Ter domain. According to the first model MatP dimers bridge two *matS* sites located on either separate chromosomes or within one chromosome. It is possible, that bridging nucleates at *matS* sites and that flanking regions are zipped up by additional nonspecific binding (and bridging) of MatP. The second model invokes an as yet unknown cofactor. After the binding of MatP, this factor would be recruited to regions surrounding *matS* sites and spread over distances up to several kb. An obvious candidate for such binding would be the H-NS protein [4] or any other NAP exhibiting cooperative binding (and bridging), but ChIP data on known NAPs do not show any evident overlap in binding patterns.

### SeqA, SlmA, MatP, and the Control of Gene Expression

As mentioned above, SeqA, SlmA, and MatP are distinct from the classical nucleoid-associated proteins in that they recognise DNA with a high degree of sequence specificity. In this respect the DNA-binding properties of SeqA, SlmA, and MatP are more akin to those of transcription factors. Intriguingly, many SeqA binding sites are located at promoters and within coding regions of genes involved in DNA replication and repair [17], and it is tempting to speculate that SeqA might regulate expression of these genes. Indeed, at some such targets (for example *mioC*, *dnaA*, *ftsZ*, *and mukB*), SeqA binding is thought to exert cell cycle–dependent control on gene expression [17], [30]–[32]. However, in other instances, SeqA binding was found to have no effect [17]. Moreover, there is little correlation between SeqA binding and changes in gene expression observed in a *seqA* mutant [17], [33]. SlmA binding sites were found mainly in coding regions of the chromosome, consistent with observations that SlmA does not appear to function as a regulator of gene expression [19], [21]. This is despite the fact that SlmA is structurally related to the TetR family of transcription factors. Similarly, whilst some MatP targets were located in intergenic regions, MatP was found to have no effect on the expression of genes in the Ter macrodomain [20]. Thus, the available data suggest that a significant proportion of binding sites for SeqA, SlmA, and MatP are not directly involved in the regulation of gene expression. Since evolution has clearly dictated that these proteins bind to specific subregions of the chromosome, we postulate that the relative positioning of SeqA, SlmA, and MatP binding sites across the genome, rather than genes targeted, is crucial. SeqA, SlmA. and MatP may act as “markers” that permit the cell to orientate chromosomes correctly, for instance, to ensure that cell division occurs where genome replication has just finished. Ultimately, detailed studies of individual SeqA, SlmA, and MatP binding loci will be required to determine the precise role of these proteins.

### Perspectives for the Future

The pattern of SeqA, SlmA, and MatP binding is probably similar among Gram negative bacteria, including the many pathogenic organisms, related to *E. coli* [17], [19], [20]. We anticipate that other proteins with macrodomain-specific DNA-binding profiles will be unearthed in the coming years. The discovery of such factors will provide new mechanistic insights into chromosome organisation, replication, and separation inside cells. The rapid detection of such proteins will require an integrated experimental approach utilizing a combination of bioinformatic, genomic, and imaging technologies. Mercier and colleagues demonstrated that careful analysis of DNA sequence can quickly pinpoint potential binding sites for proteins with macrodomain-specific DNA-binding properties [20]. Once identified such DNA sequences can be used to isolate the cognate binding factor. In this respect, recently developed “DNA-sampling” technologies, which allow the proteins bound to a specific portion of the genome to be defined, may be of particular use [34]. Currently, this approach is limited to DNA fragments a few thousand base pairs in length. However, we speculate that it may be possible to isolate individual macrodomains and apply biophysical approaches to probe their structure and protein content. Indeed, the intact nucleoid has already been purified and crudely analyzed in this way [35]. Once detected, it is essential to probe the specific role of macrodomain-associated proteins using state-of-the-art techniques, common ground already in the field of eukaryotic chromatin organisation. Specifically, detailed knowledge can be obtained using 3C-based techniques [36] that map at high resolution the spatial interaction frequencies between genomic sites. Super-resolution imaging techniques [37], [38] can provide single-cell information on the position and function of these proteins within the nucleoidal framework, as well as on spatial distance of genomic sites of interest. Finally, it is not known if macrodomains are maintained under different physiological conditions. For instance, in starved cells, the chromosome undergoes a process of super-compaction attributed to stationary phase-specific proteins Dps and CbpA [6], [39]. Drug treatment can also trigger changes in chromosome morphology [40] and this process may be particularly important for understanding the response of pathogenic bacteria to antibiotics.
